# US consumers’ mental associations with meat substitute products

**DOI:** 10.3389/fnut.2023.1135476

**Published:** 2023-03-27

**Authors:** Marion Garaus, Christian Garaus

**Affiliations:** ^1^Department of International Management, Modul University Vienna, Vienna, Austria; ^2^Department of Economics and Social Sciences, Institute of Marketing and Innovation, University of Natural Resources and Life Sciences, Vienna, Vienna, Austria

**Keywords:** meat analogs, plant-based diet, taste, health, mental associations, meat alternatives, perceptions

## Abstract

Negative impacts of meat consumption on both consumers’ health and the environment call for alternative sources for protein intake. In the last decades, the development of meat substitute products has made enormous progress. Given the beneficial aspects of reduced meat consumption, meat substitutes might be a promising approach for a more plant-based diet. However, despite the continuous improvement of meat substitute products and their increasing market potential, meat consumption in the US is still at a high level. Extant literature acknowledges that meat substitute products prompt several negative thoughts and feelings in various European countries, while US consumers’ perceptions of meat substitute products have not been investigated so far. However, understanding consumers’ thoughts and feelings toward meat substitute products provides valuable insights which can help policymakers and marketers to efficiently promote meat substitute products. Against this background, the current research investigates US consumers’ mental associations (i.e., connections of information and prior experiences with the product category stored in memory) with meat substitute products and explores if there are any differences between women and men. A sample of 175 US citizens acquired through an online panel provider completed a free word association technique resulting in 824 mental associations that qualified for the subsequent analysis. In a deductive-inductive content analysis, we assigned the mental associations to 20 categories (e.g., taste, health, environment) and determined their valence (i.e., positive, neutral, or negative). Frequencies and relationships among the categories were analyzed by employing frequency analyses, Chi-square difference tests, and multidimensional correspondence analysis. The findings reveal that meat substitute products elicit more negative mental associations than positive ones. Results validate categories identified in existing literature, but also reveal new categories of mental associations. Furthermore, the findings demonstrate that mental associations differ between women and men, with women tending to perceive meat substitutes more negatively than men. The multiple correspondence analysis resulted in four different consumer profiles (skeptics, innovators, health-oriented consumers, and avoiders) which can guide policymakers and brand managers on the effective promotion of meat substitute products.

## 1. Introduction

Despite its negative impacts on both health and the environment, global meat consumption is projected to increase by 14% by 2030 compared to the average of the baseline period 2018–2020 (1). Over the last 50 years, meat production has more than tripled resulting in more than 340 million tons of meat each year (2). This increase is well-reflected in the enormous meat market revenue. The meat market’s revenue was 1,206 bn US dollars in 2022, with an expected annual growth of 7.73%. The most portion of meat is consumed in the US, accounting for a revenue of 159.2 bn US dollars in 2022 (3). In terms of meat consumption, this relates to 224.6 pounds of red meat and poultry (4). In a global comparison, Americans are on top of the *per capita* meat consumption. On average, an American consumer eats more than three times more than the worldwide average (5). This immersive meat consumption is problematic from many different perspectives. Meat consumption is associated with several issues, such as food security concerns, animal welfare concerns, environmental concerns which relate to the depletion of natural resources, pollution, and emission of greenhouse gases, and public health concerns due to zoonotic and cardiovascular diseases (6).

In more detail, intensive meat consumption has been identified as a severe health risk. Studies confirm that meat consumption represents a risk factor for heart attack, stroke, and type 2 diabetes (7). Especially in high-income Western countries, the consumption of red and processed meat increases mortality rates at a modest level, often caused by colorectal and other forms of cancer (8, 9). Furthermore, potential explanations for these diseases refer to chemicals that are naturally contained in meat and/or released during processing and cooking (9).

Furthermore, meat products have adverse effects on the environment. Meat production signifies one of the major polluters in the food supply chain since meat requires enormous waste and causes wastewater generation and discharge (10). The meat supply chain starts with agriculture (i.e. e.g., feed storage, farm management, and primary packaging production), and ends at the final consumption stage. Between these steps, slaughterhouse activities as well as meat processing and packaging activities require additional energy resources (11). Food waste is one of the major issues in the context of meat production. Following the suggestion of Amicarelli et al. (12), food waste is considered as “food (including inedible parts) discharged, lost, degraded, consumed by pets or utilized in non-food or energy fields.” Research reveals that in Italy, from 2,678,878 t animals bred, only 1,154,393 t (i.e., 43%) are brought to the slaughterhouse. At the slaughterhouse stage, material use efficiency is estimated to be 82% (unconscious food waste), demonstrating the large potential for improvement. Additionally, only a small percentage of the energy required for beef production comes from renewable resources (a maximum of 5%). The entire Italian beef production requires approximately 11,500 t of packaging, with an energy-hidden flow (i.e., water, energy) of 1,500 TJ and more than 780,000 liters of water (11). In contrast to direct flows, which account for the actual mass of materials and hence, do not require additional material in the production chain, hidden flows (also indirect flow or embodied materials) define all materials required in the production stage for manufacturing a product (13).

On a global level, meat production is recognized as the most relevant source of methane which considerably contributes to global warming (8). Today, meat production accounts for more than half (54%) of the total emissions from agriculture (1). A recent systematic meta-analysis reviewing 369 studies report that beef and lamb meat produced the highest greenhouse gases. In comparison, field-grown vegetables account for 0.37 kg CO2-eq/kg, while beef generates 26.61 kg CO2-eq/kg and lamb meat produces 25.58 kg CO2-eq/kg (14). Among other natural sources, the production of meat requires extensive grassland which is frequently obtained by cutting down trees, causing an additional release of carbon dioxide (15).

Plant-based meat substitutes (meat alternatives, meat analogs) that describe vegetable-based food products which often include proteins from pulses, algae, cereal protein, and fungi (16), are much more sustainable than traditional meat. These plant-based products are manufactured with the overall objective to mimic aspects of meat (6, 17). As compared to conventionally produced meat, plant-based meat substitutes require significantly fewer natural resources. For instance, a beef burger causes 9.3 times more greenhouse gas emissions than a plant-based burger. Even more remarkable is the comparison of land use between plant-based and beef burgers: the latter requires 9.5 times more land use, and 546 times more water (18). In addition to these ecological benefits, the plant-based meat market has enormous growth potential. 2022, the global market value of plant-based meat reached 10.11 billion US dollars in 2022, with a predicted steady increase over the next 5 years reaching roughly 34 billion US dollars in 2027 (19).

Against the serious health and environmental consequences of meat consumption, an increase in the consumption of meat substitutes while reducing at the same time meat intake is desirable from many perspectives. A reduction in meat consumption would have a considerable positive impact on greenhouse gas emissions and health. It is predicted that a shift to a flexitarian lifestyle would reduce greenhouse gases by 583 MtCO2e per year (20) Meat substitute products are a good source of protein while at the same time, they reduce the intake of saturated fat and cholesterol as compared to meat (6). A study reports that a plant-based diet is effective in treating obesity (21) and eventually the nutritionally beneficial effects could reduce up to 52,700 premature deaths per year (20).

Despite these acknowledged positive influences, not all consumers are willing to adopt meat substitutes. Extant studies recognize the need for future research to better understand the drivers and barriers of meat substitute consumption among different consumer groups (16, 22) and the factors that encourage consumers to eat less meat (23) as well as consumers’ knowledge about the market (24). A common way to explore barriers and consumers’ expectations of plant-based meat is the assessment of consumers’ mental associations. Some prior studies have already revealed that in Germany (22), the UK and the Netherlands (13), and Portugal (25) consumers perceive meat substitute products mainly negatively. Nevertheless, knowledge of consumers’ mental associations with meat substitute products is limited, especially in the US. Given the vast amount of meat consumed in the US, knowledge of consumers’ associations with the product category meat substitute products would help policymakers and marketers to understand the drivers and barriers in the adoption process of meat alternatives. Knowledge of product categories as represented in mental associations serves as an important retrieval cue in the decision process (26), and literature recognizes the importance of food cues in determining food preferences (27). The product category itself represents an important mental association with the product (28) and as such, likely informs brand evaluations. In this context, studies verify that a strong link between the brand and the product category positively impacts memory-based brand choice (29). Against this background, the current study has the overall objective to investigate how US consumers perceive meat substitutes, and how these perceptions differ among women and men.

## 2. Literature review

This section is organized around two major themes. The first theme reviews prior studies exploring consumers’ perceptions and mental associations with meat substitute products which form the basis for the category system used in the method section. The second theme discusses the relevance of product category associations and provides theoretical arguments on the importance of exploring them in a food context.

### 2.1. Meat substitute products

Limited empirical evidence exists reporting US consumers’ mental associations with meat substitute products. A mental association is defined as a link between representations of aspects of reality or in other words, the internal cognitive structures that mirror the real world in the mind (30, 31). Individuals form mental representations about a new aspect of reality, such as a new dish, food product, or category, based on the information they gain about it and the experience they make (32). The mental representation of the new food is stored in memory along with the sensory and contextual associations. When individuals encounter the new food again, the prior mental associations such as previous judgments about the taste, texture, and smell of the product or dish are retrieved from memory as well (33). In other words, the new food serves as a stimulus that can influence the future perception of the new dish, food product, or category as well as the individual behavior (32, 33).

Studies concentrating on US samples have employed experimental designs to demonstrate that the majority (72%) of US citizens prefer a food product consisting of farm-raised beef as compared to alternative meat products (34). Other research reports that meat alternatives seem to not substitute ground meat: utilizing household scanner data, Neuhofer and Lusk (35) report that 86% of consumers who regularly consume meat substitute products consume ground meat as well. This finding was supported by Talyor et al. (36). Only 6% of the respondents acquired in a longitudinal survey (February 2020 to January 2022) indicated eating plant-based protein, while 4% indicated eating both plant-based and beef proteins on the same day. The same study collected US citizens’ perceptions of meat substitute products vs. grounded meat using closed-ended questions. Results revealed that meat substitute products are perceived to be environmentally friendly and healthy, however, they scored low on taste, price, appearance, nutrition, and naturalness (36). Other studies found that US consumers had a significantly lower likelihood of purchasing meat substitute products as compared to Chinese or Indian citizens (37). Overall, these studies agree that US citizens prefer meat over meat substitute products, however, consumers’ mental associations with meat substitutes have not been investigated that far. In support of this notion, a recent literature review on consumers’ mental associations with food consumption did not identify any study conducted in the US (38).

Although to the best of our knowledge, no study exists which explores US consumers’ mental associations with meat substitute products, some studies provide valuable insights into consumers’ mental associations with meat substitute products in Europe. First exploratory studies found that two major drivers prompt consumers to consume meat products, namely ecological welfare and political values (16, 39). However, on a general level, it seems that meat outperforms meat substitutes in terms of positive mental associations. A study with a sample from the UK and the Netherlands reveals that meat is associated with good health and mood, convenience, sensory attractiveness, and luxury. On the contrary, only a few positive mental associations were identified for meat substitutes, namely ethical aspects and weight control (16). Similar results were obtained by a study conducted in Germany. The most frequent mental associations with meat substitute products were “tofu”, “vegan”, and “disgust”, while meat products prompted the mental associations “delicious,” “food,” and “taste” most frequently (22). Supporting these findings, a study conducted in Scotland reports that various alternatives of meat substitutes (tofu, seitan, legumes, insects, and lab-grown meat) prompt feelings of disgust (25). Whereas UK respondents indicated a considerably lower utility level for meat substitutes than any other type of meat, there are certain attributes with high utility levels. Country of origin, low-fat content, and low carbon footprint are positively associated with meat substitute products and hence might be fruitful as a promotion strategy (40). In support of these findings, organic and local represent important attributes predicting the choice for meat substitute products based on micro-algae (41).

Other research employing closed-ended questions report that ethical concerns are a strong driver for willingness to eat cultured meat in Germany, while perceptions of unnaturalness and potential damage to farmers represent negative drivers (42). Unnaturalness has also been identified as a barrier to meat substitute adoption in the US, in addition to the limited taste and appeal (43). On the one hand, health and sustainability aspects have been identified as the main drivers for consumers’ intention to consume meat substitute products in China (39). On the other hand, other research revealed a lack of awareness of the mental association between meat consumption and climate change (44). In depth interviews with 20 Dutch consumers revealed mainly positive or neutral mental associations with meat substitutes, such as “traditional meat replacement,” “nutrition substitution” or “specific meat substitute products” (45). Interestingly, there was no consensus on the requirements for meat substitute products, some respondents indicated that meat substitute products should be similar to real meat while others held the opposite opinion. It needs to be mentioned that all of the 20 respondents were familiar with meat substitute products and some of them were vegetarian (45). However, other studies acknowledged the challenge to attract not only vegetarians but people who regularly eat meat (16). Given the low proportion of 1–2% of vegetarians of US citizens (6), it is important to collect mental associations of the broader population.

Related to this, some demographic variables have been identified to impact consumers’ mental associations with meat substitute products. For instance, a positive impact of education level on meat substitute consumption was observed. Individuals with higher education consumed significantly more meat substitute products in the UK and the Netherlands (16). The mental associations with meat alternatives in the UK and Netherlands did not differ between gender (22). Nevertheless, another study reported differences in motives for eating meat substitute products between men and women. Women tend to base their decision to eat meat alternatives on sustainability reasons and health concerns, while additionally, other personality characteristics, such as low disgust sensitivity, higher education, and lower age are additional drivers of meat substitute consumption (46). Interestingly, men in the US seem to be more willing to switch to another meat alternative—*in-vitro* meat—on a regular basis as compared to women (43), which is why a higher intention to change could be assumed for men. On the contrary, a comprehensive literature review reveals that men in general consume more meat and have a lower willingness to eat plant-based meals (47). Similarly, educational status predicted the willingness to follow a plant-based diet (47). Hence, educational status as well as gender seem to influence consumers’ mental associations with meat substitute products. In order to gain a better understanding of the importance of product category associations, and their potential to transfer any thoughts and feeling to a brand belonging to this product category, the next section elaborates on the formation of category-based brand associations.

### 2.2. Product category associations

Meat substitutes represent a new product category, including several brands, such as Impossible Foods, Beyond Meat, Gardein, and Amy’s Kitchen. Rather than exploring the brand associations on a brand level, the current research concentrates on mental associations with the product category (i.e., a particular group of related products) (48). Mental associations with product categories are an important predictor of brand associations, and brand associations play an important role in consumers’ product evaluations and choices (49). Brand associations describe any type of information that is linked to the brand node in memory, such as the product category itself, the usage context, or any other evaluative thoughts (e.g., taste, texture) (50, 51). The allocation of a brand to a specific brand category is the first step in building strong brands (52).

Extant research acknowledges the relevance of categorization in the context of meat substitute products (53). In 2011, consumers merely associated meat substitute products as different as compared to processed meat substitute products, while some product categories (e.g., sausages, burger patties) included both, meat and meat substitutes (53). The authors call for research that explores the underlying attributes which determine the product category classification. Product classification is important for a brand to make it into the awareness and evoked set during the purchase process (54) and for the identification and differentiation of brands (55). There is consensus in extant literature that each product category has its own specific mental associations (56).

Schema theory represents a theoretical explanation for the relevance of product categories and their corresponding mental associations. In the branding context, schemas are cognitive structures that define the expectations of specific product categories in the form of values on attributes, the weight of these attributes, and the variability across different brands (57). If consumers encounter a new product or brand, they will first process the product category knowledge to which this product or brand belongs. Research confirms that category schemas influence consumers’ responses to local and global brands (58). Other studies show that products need to match the category color norms in order to prompt favorable attitudes and purchase intention (59). Individuals compare a stimulus (brand) to the exemplar (product category) and if the stimulus fits the exemplar, an affective transfer occurs (60). In other words, in such a category-based judgment, the thoughts and emotions are transferred from the product category to the brand. In the context of meat substitutes, research reveals that not taste itself, but the symbolic meaning associated with the product category of meat substitutes determines taste evaluations. The authors conclude that heavy meat eaters should be addressed with values they endorse when promoting meat alternatives (60). However, if a product does not fit a product category by threatening existing beliefs, consumers most likely react negatively (61). Hence, it is important to know which favorable mental associations exist for meat substitutes. At the same time, knowledge of potential negative mental associations would provide new knowledge on barriers to meat substitute consumption and might guide manufacturers on strategies aiming at eliminating these negative mental associations.

## 3. Materials and methods

### 3.1. Sample selection

To answer our research questions, we drew on an online sample acquired through the online panel provider “Clickworker” in September and October 2022. An invitation to participate in our study was posted on Clickworker’s web platform and respondents were offered a small compensation for their participation. Only US citizens were allowed to participate in our study. As is typical with online panel providers, potential participants decided for themselves whether they wanted to participate in our study. Because the resulting self-selection bias is an issue in online panels, our sampling strategy was non-probabilistic (62). While the non-probabilistic samples are unlikely to be perfectly representative of the target population, they are particularly useful for qualitative methods (63) such as the one employed in our study. For a detailed discussion of the advantages and disadvantages of non-probabilistic samples for qualitative studies see Patton (63).

### 3.2. Data collection

To collect the data, we applied the free word association technique, which is particularly suited to studying the structure of mental representations (32). The idea underlying the free word association technique is that the responses that are triggered by an unstructured and ambiguous stimulus, elicit the participants’ deep feelings, beliefs, and attitudes (37). Following the steps in the procedure of the free word association technique, we first familiarized the participants with the task and assured them that there are no right or wrong answers. We also instructed them to enter only single words or expressions. Then, we asked them to list those words that spontaneously come to their mind when we presented them with the stimulus: “Please let us know what you think about meat substitute products (e.g., meat-free minced meat, meat-free sausages).” Afterward, participants could enter their verbal responses into open text boxes in a questionnaire that we created using the web application “SoSci Survey.” We asked the respondents to write down as many words as came to their minds. We deliberately did not limit the number of words and provided them with unlimited time to not restrict the thought process (32).

Once participants had shared all of their thoughts and feelings about meat substitutes in the free word association technique (one open question), we collected several demographic and sociodemographic data. In specific, we asked respondents to indicate their answers for the variables age (one item), gender (one item), educational attainment (one item), ethnicity (two items), employment status (one item), and personal income (one item).

It is important to mention that the study was conducted in accordance with the revised version of the Declaration of Helsinki and was approved by the first author’s institutional review board. Informed consent was obtained right on the first page of the questionnaire. More specifically, the following statement was included “Your participation is voluntary and you are free to withdraw at any time, without giving a reason and without cost. By clicking the “Next” button, you voluntarily agree to take part in this study.” Respondents who failed attention checks (please tick the middle of the scale) were automatically excluded from the survey.

### 3.3. Data analysis

The data were analyzed using a deductive-inductive content analysis. Deductive-inductive approaches combine the strengths of deductive and inductive content analysis (64). In the first step, deductive categories are developed based on existing literature. Each category is precisely defined, clear coding rules are established (i.e., when a text passage is assigned to that category), and reference examples are given (65). Once the resulting category system is finalized, the actual coding takes place, in which passages are assigned to the categories. In the second step, inductive categories are developed from the text material that contains new aspects that have not yet been adequately described in the existing literature and thus, could not be assigned to an existing category. To code these new aspects, new categories are formulated based on the key information contained in the text (64). In the final step, these categories are revised and integrated into the category scheme.

We followed this deductive-inductive procedure by first deductively developing categories based on extant literature exploring mental associations with meat-substitute products. Starting with this preliminary category scheme, we coded the data. All mental associations were assigned to these semantic categories by the two researchers. Inconsistencies were discussed until a consensus regarding a specific category was reached. In the process of coding the data, not all mental associations could be categorized, requiring the inductive creation of new categories.

**Figure 1:**
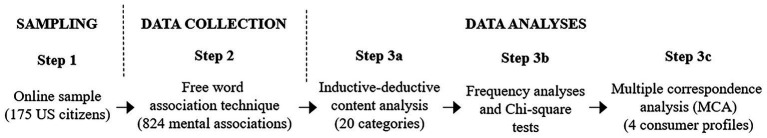
Overview of the methods applied for sampling, data collection, and data analyses.

Once our category scheme was complete, we performed frequency analyses of the categories. Furthermore, we investigated differences between gender using Chi-square tests. For a deeper analysis, we then conducted a Multiple Correspondence Analysis (MCA). MCA is an exploratory multivariate technique of data analysis (66). It is used to study relationships between categorical (nominal) variables and graphically represent them in the form of a biplot, which makes it a useful tool for analyzing free word associations (67). When used to analyze data on free word associations, each point represents a category of mental associations and the position of the points in the biplot reflects the relationship with other categories. MCA thus enables the identification of patterns and clusters within categorical data. For this reason, we analyzed our study participants’ positive and negative mental associations with meat substitute products using MCA. The multiple correspondence analysis represents the final step in our data analysis. For a better overview of all methodological steps in our study, we have summarized the methods of sampling, data collection, and data analysis in Figure 1.

## 4. Results

### 4.1. Sample characteristics

175 participants completed the survey. In the first step, the data were cleaned from non-meaningful terms, leading to an exclusion of three respondents. As a result, a total of 824 mental associations elicited by 172 participants qualified for the data analysis. Participants were between 18 and 66 years old, with an average age of 36.4 years. Among the participants, 59.9% were female and 34.3% were male, 1.7% were transgender, and 4.1% did not answer this question. As regards the highest educational attainment, 46.5% had an advanced or bachelor’s degree, 29.7% held a college or associate degree, 18.6% finish high school, and 1.2% did not complete high school. The majority of participants were employed, with 40.1% working full time, 5.8% with a part-time employment, and 16.3% being self-employed. 5.2% were students, 8.1% indicated “homemakers” as employment status, and 3.5% preferred not to stated their employment status. Participants’ income was assessed in eight categories, whereas 3.5% indicated to have no income at all, 25% earned between $1 and $9,999, 13.4% had an income between $10,000 and $24,999, 23.8% confirmed to have an income between $25,000 and $49,999, 8.7% indicated to earn between $50,000 and $74,999, 7.6% received an income between $75,000 and $99,999, 6.4% earned between $100,000 and $149,999, and finally, 2.9% reported to earn $150,000 and greater (8.7% did not report their income). Hence, about two-thirds of the sample earned less than the median income. Most respondents were White, not Hispanics (59.9%) and White Hispanics (11.6%) followed by African American (7.6%), Asian (5.8%), Native American (4.1%), and Others (2.9%).

The distribution of ethnicities in our sample closely mirrors the US population. In terms of employment, those who work full-time are also comparable to the US population. Our sample is also broad and diverse for the other sample characteristics. However, as common for online samples, our participants are more often self- or unemployed, tend to be younger, and are better educated (68). Women and transgender persons are overrepresented in our sample, and their income is lower. Approximately two-thirds of the sample earns less than the median income.

### 4.2. Categorization of mental associations

Three new categories emerged during the coding process: “innovation,” “nutrients,” and “additives.” The cycling between the data and the coding scheme also resulted in the deletion of the preliminary categories “mood,” “ethics,” “social influence,” “risk,” “Should not be similar to meat,” “Should be similar to meat,” “Not all are good,” and “satiety,” because they were not mentioned. In addition, the sensory appeal category was divided into more specific categories, namely “general appeal,” “taste,” “texture,” and “smell.” Furthermore, the preliminary categories “alternative protein foods” and “specific meat substitutes” were renamed into “protein sources” and “specific brands,” respectively to better reflect the customers’ knowledge about specific brands or protein sources as represented in meat substitute products. Multiple mental associations from one person that could be classified into the same category were counted as one, resulting in 437 distinct case-category codes. For further analysis, the mental associations were further split up into mental associations related to diet (Table 1) and mental associations related to motives for meat substitute consumption (Table 2). For the motives for meat substitute products, the mental associations were further classified into positive or negative mentions to allow a distinction between positive and negative mentions. This step resulted in 136 neutral mentions and 301 with positive or negative valence. The analysis proceeded with an investigation of the most frequent mental associations, the portion of positive and negative mental associations as well as differences between women and men.

**Table 1:** Mental associations related to diet.

Mental associations related to diet	Exemplary mental associations
No meat	Meat-free, plant-based, sourced from plants
Meat products	Beef, pork, chicken
Protein sources	Pea protein, soya protein, lupin protein
Vegetables	Potato, vegetable, fruit
Meat substitute brands	Beyond Meat, Impossible Burger, MorningStar Farms
Meat substitute products	Meat-free minced, meat-free sausages, veggie burgers
Meat replacement	Substitute, switch
Vegetarian/vegan diet	Vegetarian-friendly, vegan-friendly, vegan eating

**Table 2:** Mental associations reflecting motives and barriers for meat substitute consumption.

Mental associations related to motives	Exemplary positive mental associations	Exemplary negative mental associations	Frequency of mental associations	
			Pos.	Neg.
Taste	Tasty, yummy, delicious	Tasteless, bland, bad flavor	19	28
Health	Healthy, weight control, longer life	Unhealthy, stomach aches, cancer-causing	32	11
Disgust	n.a.	Gross, nasty, Eww, Yuck	0	24
Fake	It’s real	Fake, misleading, deceptive	1	18
General appeal	Appetizing, mouth-watering	Unattractive, unappealing, unappetizing	2	16
Innovation	Innovative, new, unique	n.a.	16	0
Skepticism	Reliable, trusting	Weird, strange, questionable	1	15
Environment	Eco-friendly, planet-friendly, combats climate change	n.a.	14	0
Texture	Toothsome, satisfied with the consistency	Gritty, rubbery, chewy	2	12
Price	Cheap, cheaper	Expensive, pricey, overpriced	2	11
Natural content	Natural	Unnatural, against nature, lab-made	1	10
Curiosity	Curious, interesting	Uninterested, I do not care	8	3
Familiarity	Know	Unknown, Idk, not formed opinion	1	7
Animal welfare	Cruelty-free, less animal cruelty	n.a.	7	0
Additives	Clean, free	Additive, preservatives, meat glue	4	3
Variation	Versatile	Average	3	2
Convenience	Easy, quick, convenient	n.a.	3	0
Smell	n.a.	Stinky, odd smell	0	2
Nutrition	n.a.	High sodium	0	3
Other	Good, improved, feasible	Bad, waste, depressing	12	11

### 4.3. Frequencies of mental associations with meat substitutes

The three most frequent positive mental associations are “healthy”, “tasty”, and “innovative”. Three most frequent negative mental associations are “not tasty”, “disgusting”, and “fake”. Some categories feature only positive but no negative mental associations. For instance, the new category “innovation” prompted only positive mental associations. Likewise, there was general broad agreement that meat substitute products are good for the environment, while no participants verbalized thoughts that meat substitutes could harm the environment. On the contrary, several consumers perceived meat substitute products to be unnatural, while only one respondent associated meat substitutes with “natural.” Meat substitute products are often perceived as pricy. Eleven price associations were “expensive”, while only two were “cheap”. Several positive and negative mental associations were classified into the health category. In this category, an interesting pattern was observed: the positive mental associations in the health category were quite unspecific (e.g., “healthy,” “nutritious,” “longer life”), while negative mental associations are specific and seem to focus on short-term consequences (e.g., “stomach aches,” “heartburn,” “diarrhea”) as well as medium-term consequences (e.g., “anemia”), and long-term consequences (e.g., “cancer-causing”). Mental associations which expressed respondents’ general attitude toward meat substitute products, either positively (e.g., “great,” “decent,” “useful”), neutrally (e.g., “fact,” “different,” “recipes”) or negatively (e.g., “bad,” “waste,” “depressing”) were classified into the category “other.” The frequencies of the positive, neutral, and negative mental associations in the category “other” were about the same.

### 4.4. Valence and gender differences of mental associations with meat substitutes

Overall, more negative (56%) than positive (44%) mental associations were observed. There was a remarkable difference in positive and negative mental associations between women and men though. Men had much more positive mental associations (63%) than negative ones (37%), while the opposite was true for women, who had 33% positive and 67% negative mental associations. Additional analyses (Chi-square tests) provided more detailed insights into the specific mental associations causing this significant difference. Women associated meat substitutes significantly more often with “vegan” or “vegetarian” (17%), while this mental association was not elicited by men [only 3%; *Χ^2^*(1, 162) = 6.88, *p* = 0.01]. On the contrary, men associated meat substitute products more often with tastiness (20%), while only 5% of women mentioned this positive mental association [*Χ^2^*(1, 162) = 9.58, *p* = 0.00]. Furthermore, women considered meat substitute products more often as fake (15%) than men [3%; *Χ^2^*(1, 162) = 4.99, *p* = 0.03]. Also, in the “others” category, women had more negative mental associations (10%) than men [0%; Chi-square test *Χ^2^*(1, 162) = 6.11, *p* = 0.01]. These gender differences in mental associations with meat substitute products were also reflected in the MCA as we will elaborate on below.

**Figure 2:**
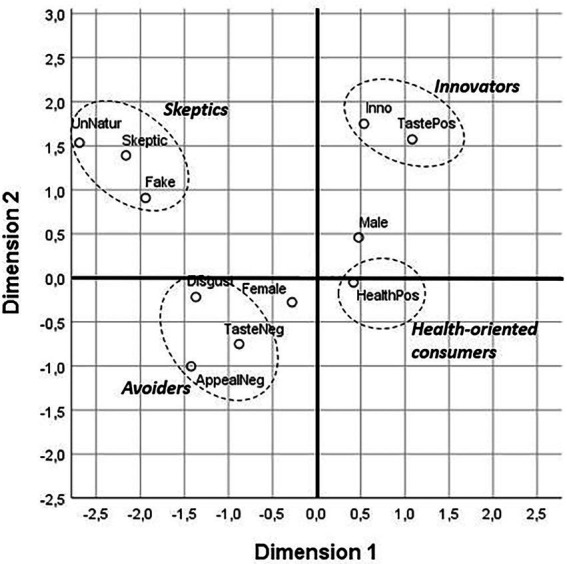
Biplot of the relationships between mental associations with meat substitutes.

We conducted the MCA to identify profiles of individuals based on their mental associations related to motives for meat substitute consumption (Table 2). Following the suggestion of Sester et al. (46) we utilized only categories mentioned by more than 5% of mental associations (minimum threshold = 15) for the MCA. The MCA revealed two dimensions: the first dimension with an inertia of 23.2%, and the second dimension with an inertia of 13.8%. The biplot, which depicts a topological representation is illustrated in Figure 2. The two diagonals divided people in terms of (1) taste perceptions and (2) healthy/unnatural perceptions. The first diagonal classifies consumers into individuals who appreciate the taste of meat substitutes vs. individuals who do not like the taste of meat substitutes and do not experience them as appealing. The second diagonal classifies individuals that perceive meat substitute products to be healthy vs. those that consider them unnatural. The latter individuals had mental associations such as “overprocessed,” “artificial,” and “lab-made.”

Four different consumer profiles were identified. The first profile, which we call “skeptics” consists of individuals that are skeptical and consider meat substitutes as unnatural or even fake. The second profile, which we call “innovators” includes individuals that perceive meat substitutes as innovative and tasty. The third profile, which we call “health-oriented consumers” includes individuals that consider meat substitutes as healthy. Finally, the fourth profile, which we call “avoiders,” reflects individuals that perceive meat substitutes as distasteful and unappealing, and even feel disgusted.

With regard to gender, we again observed differences. From Figure 2, it can be seen that the barycenters of both genders are on the tasty/untasty diagonal. Women are located near profile 4 (“avoiders”) close to the barycenter of the negative mental associations with “taste,” while the barycenter of men is between profile 2 (“innovators”) and profile 3 (“health-oriented consumers). We turn to the interpretation of these results in the next section.

## 5. Discussion

Despite alarming obesity figures, the detrimental effect of immoderate meat consumption on health as well as the negative consequences of excessive meat production on the environment, only a minority of US citizens follows a plant-based diet. While there are three different ways to reduce meat consumption— (1) eat less but higher quality meat, (2) replace animal proteins with plant-based foods that have no similarities to meat (e.g., beans), (3) consume meat substitute products that mimic meat—our study focused on the last of these ways. Since meat substitute products can often substitute meat one-to-one in conventional dishes, consuming them is a particularly easy way to promote healthier and more sustainable eating habits. Nevertheless, little is known about the mental associations with meat substitute products among US citizens. The current study set out to fill this gap. It employed a free word association task and surveyed 175 US citizens on their mental associations with meat substitutes. Product categorization is important in the context of meat substitute products (52) and enables marketers and researchers to understand how consumers perceive these new products. Brand managers need to be aware of consumers’ mental associations with the product category to effectively promote brands belonging to this product category (54, 55). The data analysis is based on 824 mental associations. Frequency analyses and a multiple correspondence analysis reveal new and interesting insights into US consumers’ mental associations with meat substitute products.

Overall, the findings indicate that US citizens have more negative mental associations with meat substitute products than positive mental associations. This finding expands prior research reporting that consumers have more positive mental associations with meat as compared to meat substitute products (13), while our study concentrates only on meat substitutes. In contrast to prior studies, our findings further demonstrate that gender represents an important variable in explaining consumers’ positive and negative perceptions of meat substitute products. In doing so, we advance existing research reporting inconclusive findings on differences in men’s and women’s mental associations with meat substitute products.

Mental associations with meat substitutes differ between men and women. It seems that women consider meat substitute products part of a vegan or vegetarian diet, and not as an alternative source of protein that could substitute meat occasionally. Women seem to have rather utilitarian associations, by concentrating on the nutritional aspect rather than on hedonic associations. This pattern has been observed in prior research as well (46). Indeed, women associated meat substitute products with a bad taste and disgust, mental associations that have been reported by prior studies employing non-US samples as well (25).

In this context, the unhealthy-tasty intuition might explain the prevailing negative mental associations of women with meat substitute products (69). In essence, the unhealthy-tasty intuition postulates that individuals associated unhealthy products with good taste, while the opposite is true for healthy products (69). The unhealthy-tasty intuition has been confirmed in various contexts. For instance, one study validates the unhealthy-tasty intuition in the context of recipes by reporting that a health claim negatively affects taste expectations (70). Hence, if meat substitute products prompt merely nutritional associations together with health inferences (as revealed in the MCA), women might draw the conclusion that meat substitute products are healthy but at the same time, also less tasty. Nutrition information indeed prompts more health inferences than taste inferences. A recent study demonstrates that presenting nutrition information before tasting plant-based products causes consumers to pay more attention to health inferences rather than taste inferences (71). Furthermore, women tend to perceive meat substitutes as fake products and are skeptical of these products. One possible explanation for these mental associations is that women are in general responsible for the nutrition of the family and hence, more cautious (72). Furthermore, these mental associations might be caused by little awareness of the benefits of meat substitute products. Prior research acknowledges that a lack of knowledge on how a plant-based can positively impact health and the environment represents a major barrier to plant-based diets (73).

On the contrary, men experience meat substitute products as innovative, which at the same time also represents one of the new categories that emerged in this study. Indeed, prior research has not identified “innovation” as an important mental association with meat substitute products. However, given the trend of variety seeking (74), new and innovative products seem to prompt favorable mental associations, while this mental association is predominantly elicited by men. Men associated meat substitute products with good taste and had fewer negative mental associations as compared to women. These findings contribute to the debate on men’s willingness to consume plant-based meals (47) and potentially also the related research streams on alternative proteins such as *in-vitro* meat (43).

Overall, the study reveals four consumer profiles. The first profile represents the “skeptics,” who mistrust information about meat substitute products and who experience them as fake and unnatural. The second profile is the “innovators,” who have a generally positive attitude towards meat substitute products and associate them with good taste. The third profile is the “avoiders,” who experience meat substitute products as unappealing, disgusting, and unappealing. Finally, the fourth profile represents the “health-oriented consumers,” who consider meat substitutes as a good alternative for a healthier (vegan or vegetarian) diet. While the profile of health-oriented consumers has also been found in European studies and also the “avoiders” profile was similarly described by Possidonio et al. (25), US citizens still seem to differ in their mental associations. Ethical considerations play a subordinate role, while the novel character of meat substitutes was clearly more important as the profile of the “innovator” demonstrates. Also, the profile of “skeptics” is interesting, as it has not been observed in this pronounced way by prior studies.

When these profiles are combined with the genders, interesting patterns can be observed. Women seem to be best represented by the profile “avoiders,” while men’s mental association justified a classification into the profile “innovators.” These new insights have several important practical implications: Policymakers and brand managers of meat substitute products need to have different targeting strategies based on gender. Men’s consumption of meat substitute products could be stimulated by highlighting the innovative character and the good taste of the products. For women, a focus on the elimination of negative mental associations by providing more information on the processing of meat substitutes might be a good strategy. In general, reducing skepticism toward meat substitute products through governmental campaigns might be a fruitful policy, which should mainly target women. Additionally, highlighting good taste seems to be a good strategy for all consumer profiles, since taste represents one of the most important predictors of food consumption (75).

## 6. Conclusion

In conclusion, plant-based diets are beneficial for individuals and society. The current research contributes to the ongoing discussion on consumers’ mental associations and perceptions of meat-substitute products in an effort to provide new insights which help both policymakers and marketers to better promote meat-substitute products. In addition to the identification of gender differences in terms of mental associations with meat substitute products, the current study identifies four different consumer profiles, which can be used for targeting purposes. Further studies might test specific promotional strategies (i.e., taste vs. health claims) for the identified consumer profiles.

One of the strengths of our study is that we discovered gender differences in mental associations with meat substitutes. Future studies could collect other person-specific data, such as actual meat consumption, and relate it to mental associations with meat substitutes.

Although our sample was large and diverse, it is an online sample and thus limited in its representativeness (e.g., concerning age or educational attainment). Thus, further work could use other sampling strategies that allow for examining our findings in larger and more representative samples to validate the results for the U.S. population. Another promising avenue is to replicate our study in other countries to compare the mental associations with meat substitutes across cultural contexts (37).

Our research could present the starting point for future research. On the one hand, additional qualitative work could be conducted using projective techniques (e.g., construction, completion, order of choice, or expressive) to make systematic comparisons between the different methods of analysis. On the other hand, quantitative work could develop scales to gain additional information about consumers’ perceptions of meat.

## Data availability statement

The raw data supporting the conclusions of this article will be made available by the authors, without undue reservation.

## Ethics statement

The studies involving human participants were reviewed and approved by Institutional Reviewer Board of Modul University Vienna. The patients/participants provided their written informed consent to participate in this study.

## Author contributions

MG and CG: conceptualization, data curation, and writing – review and editing. CG: methodology and formal analysis. MG: writing – original draft preparation and project administration. All authors contributed to the article and approved the submitted version.

## Funding

This research was supported by the Oesterreichische Nationalbank (Anniversary Fund, project number: 18757).

## Conflict of interest

The authors declare that the research was conducted in the absence of any commercial or financial relationships that could be construed as a potential conflict of interest.

## Publisher’s note

All claims expressed in this article are solely those of the authors and do not necessarily represent those of their affiliated organizations, or those of the publisher, the editors and the reviewers. Any product that may be evaluated in this article, or claim that may be made by its manufacturer, is not guaranteed or endorsed by the publisher.
